# A118 BALLOON-ASSISTED ENTEROSCOPY TIMING INCREASES DIAGNOSTIC YIELD IN PATIENTS WITH OVERT OBSCURE GASTROINTESTINAL BLEEDING: A RETROSPECTIVE STUDY

**DOI:** 10.1093/jcag/gwac036.118

**Published:** 2023-03-07

**Authors:** J Guo, G Malik, S Wasilenko, B Halloran, A J Montano-Loza, S Zepeda-Gomez

**Affiliations:** Division of Gastroenterology, Department of Medicine, The University of Alberta, Edmonton, Canada

## Abstract

**Background:**

Obscure gastrointestinal bleeding (OGIB) is defined as bleeding from an unknown etiology despite initial investigations with upper endoscopy and colonoscopy, of which 75-80% is attributed to a small bowel (SB) source. OGIB poses a significant diagnostic and therapeutic challenge, resulting in high morbidity and mortality with increased utilization of health care resources. Balloon-assisted enteroscopy (BAE) is a useful procedure for the evaluation and management of small bowel bleeding, with reported diagnostic and therapeutic rates up to 87% and 80%, respectively.

**Purpose:**

This retrospective study aims to evaluate the diagnostic and therapeutic yields in a large cohort of adult patients presenting with different subtypes of OGIB who have undergone BAE, as well as to assess for association between various patient and disease factors, and clinical outcomes.

**Method:**

We performed a retrospective review of 1057 cases of BAE at a large quaternary referral centre between 2016 to 2021 and 158 OGIB cases were identified. Sex, age, and, preprocedural variables including indication, time from video capsule endoscopy (VCE) to BAE and subclassification of SB bleed were collected. Endoscopic modality, findings, and therapeutic interventions were used to calculate diagnostic and therapeutic yields. The association between the timing of BAE relative to VCE and clinical outcomes including rebleeding rate, diagnostics and therapeutic yields were assessed. Bleeding free-survival was estimated using Kaplan-Meier function. All data analyses were performed with SPSS.

**Result(s):**

The overall diagnostic yield of BAE was 74%. Patients with active overt bleeding were found to have a statistically significant higher yield compared to those with inactive overt bleeding (94% vs 67%, P= 0.03). The therapeutic yield was 51%, with a significantly higher rate of injection therapy and hemoclip placement in those with active overt bleeding compared to those with inactive overt and occult bleeding (P< 0.001). BAE performed within 72 hours of overt GI bleeding was found to have a statistically significant higher diagnostic yield when compared to procedures performed after 72 hours (94% vs 67%, p =0.05). Univariable analysis revealed higher diagnostic yield in patients requiring transfusion within the past 12 months (P= 0.02) Finally, rebleeding-free survival in all patients at 1 year and 5 years was 98% and 68%, respectively.

**Image:**

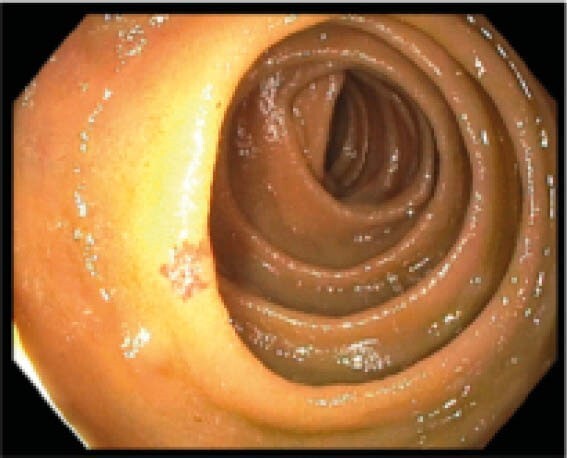

**Conclusion(s):**

Balloon-assisted enteroscopy continues to be an effective diagnostic and therapeutic modality for the investigation of obscure gastrointestinal bleeding. Our retrospective study shows a higher diagnostic yield in those presenting with active overt GI bleed as well as those who underwent BAE within 72 hours of overt GI bleeding. Furthermore, patients who required transfusion within the past 12 months are more likely to have a positive BAE finding.

**Please acknowledge all funding agencies by checking the applicable boxes below:**

None

**Disclosure of Interest:**

None Declared

